# Research progress of rebound pain after nerve block in arthroscopic rotator cuff repair

**DOI:** 10.3389/fmed.2025.1659133

**Published:** 2025-10-15

**Authors:** Xue Yang, Yujiao Yang, Sulan Qin, Yunliang Chen

**Affiliations:** ^1^Clinical Medical College of North Sichuan Medical College, Nanchong, Sichuan, China; ^2^Department of Anesthesiology, Guang'an People's Hospital, Guang'an, Sichuan, China

**Keywords:** arthroscopic rotator cuff repair, nerve block, rebound pain, postoperative pain, postoperative analgesia

## Abstract

Arthroscopic rotator cuff repair (ARCR) is a commonly performed surgical intervention for rotator cuff injuries. The advent of ultrasound guidance has facilitated the widespread adoption of nerve blocks as an adjunct anesthetic and analgesic strategy in the perioperative phase of ARCR, offering notable benefits in maintaining patients' intraoperative hemodynamic stability and reducing opioid consumption. Despite the minimally invasive nature of ARCR, a subset of patients still experiences substantial pain, encompassing both acute pain and rebound pain following nerve block. Post-nerve block rebound pain is characterized by severe and disabling discomfort, which adversely impacts the patient experience and postoperative recovery. The mechanism underlying rebound pain after nerve block may involve aberrant excitation of relevant nerve fibers, the pharmacology of local anesthetics, nerve injury, local inflammatory factors, surgical anesthesia, and patient-specific variability. The review suggests that the incidence of post-nerve block rebound pain can be reduced, and postoperative pain management outcomes following ARCR can be improved, through interventions such as continuous nerve block, combined peripheral nerve block, administration of local anesthetic adjuvants, multimodal analgesia, and patient education.

## 1 Introduction

Rotator cuff injuries represent a common source of shoulder pain in middle-aged and elderly populations, primarily resulting from soft tissue damage to the tendons of the subscapularis, supraspinatus, infraspinatus, and teres minor muscles surrounding the glenohumeral joint. Clinically, these injuries are characterized by shoulder pain and restricted range of motion, potentially impacting daily function and quality of life (1, 2). In the general population, the overall prevalence of rotator cuff injuries is estimated at 20%−40%, rising to 50%−60% among individuals aged 60 years and above. Arthroscopic rotator cuff repair (ARCR) serves as a primary surgical intervention for rotator cuff injuries, offering effective pain relief and functional improvement (3). Despite its minimally invasive nature, ARCR is associated with varying degrees of postoperative pain, and the incidence of severe intraoperative or postoperative pain has been reported to be as high as 45% (4). In recent years, ultrasound-guided nerve blocks have become a common adjunct to anesthesia and analgesia during and after ARCR, owing to their superior analgesic efficacy and minimal systemic side effects. This technique offers effective perioperative analgesia, lowers opioid consumption and associated side effects, and may reduce hospital stay duration (5). However, significant pain may occur in some patients following the resolution of the nerve block, potentially due to rebound pain. Rebound pain, a significant complication after nerve block, is associated with a high incidence and may require increased postoperative opioid use, thereby diminishing the overall benefits of nerve block (6). This review aims to examine the definition, clinical characteristics, underlying mechanisms, influencing factors, and management strategies related to rebound pain following nerve block. The objective is to provide a framework and clinical guidance to mitigate rebound pain after an ARCR nerve block and optimize postoperative pain management protocols.

## 2 Material and methods

A literature search was performed in the PubMed, Web of Science, and China National Knowledge Infrastructure (CNKI) databases, with studies identified up to June 2025. The following search terms were employed: “arthroscopic rotator cuff repair,” “shoulder arthroscopy,” “rotator cuff injury,” “nerve block,” “rebound pain,” “postoperative pain,” “intermuscular groove brachial plexus nerve block,” “multimodal analgesia,” and “continuous nerve block.” Relevant literature, including reviews, clinical trials, randomized controlled trials, case reports, systematic reviews, and meta-analyses related to nerve block, rebound pain, postoperative pain, and analgesia following ARCR, was considered for inclusion. Irrelevant, duplicate, or controversial studies were excluded. The sources and content were independently screened by two researchers, and a total of 77 studies were ultimately selected for analysis.

## 3 Definition and clinical features of rebound pain after nerve block

The definition of rebound pain following nerve block remains without a universally accepted standard. In 2007, Williams first introduced the term “rebound pain” after nerve block to describe the acute and severe pain that occurs as the effects of the nerve block dissipate (7). In this study, rebound pain was operationalized as the measurable difference between the lowest pain score in the initial hours after block resolution, proposing the “rebound pain score” to quantify it, calculated as the highest pain score within 12 h of block resolution minus the lowest pain score before block resolution (7). Shi et al. (8) and Li et al. (9) used “rebound pain” as a translation of “flare pain.” Stone et al. (10) recommended a more neutral term, such as “pain on block resolution,” pending further clarification of its etiology and clinical implications. Given the current diversity of terminology used in academic literature to describe the acute pain phenomenon following the dissipation of nerve block effects, this review adopts “rebound pain” as the standardized term to maintain consistency and precision in its discussion. It is explicitly defined as acute, intense pain that emerges following the complete restoration of sensory function after nerve block. This definition and terminology will be uniformly applied throughout the text to ensure academic rigor and logical consistency.

Clinically, post-block rebound pain after ARCR typically occurs at night, begins 12–24 h post-nerve block, lasts 2–6 h, and progressively escalates, culminating in peak state nociceptive hypersensitivity. Patients often described the pain as a burning or dull ache in the shoulder. Individual variability in nerve block regression makes it difficult to determine the precise onset of rebound pain (11). Current research suggests a strong correlation between rebound pain intensity and the inflammatory repair process around the surgical site, indicating that it is a transient pain response distinct from chronic postoperative pain (12).

## 4 Mechanism of rebound pain after ARCR nerve block

**Figure 1:**
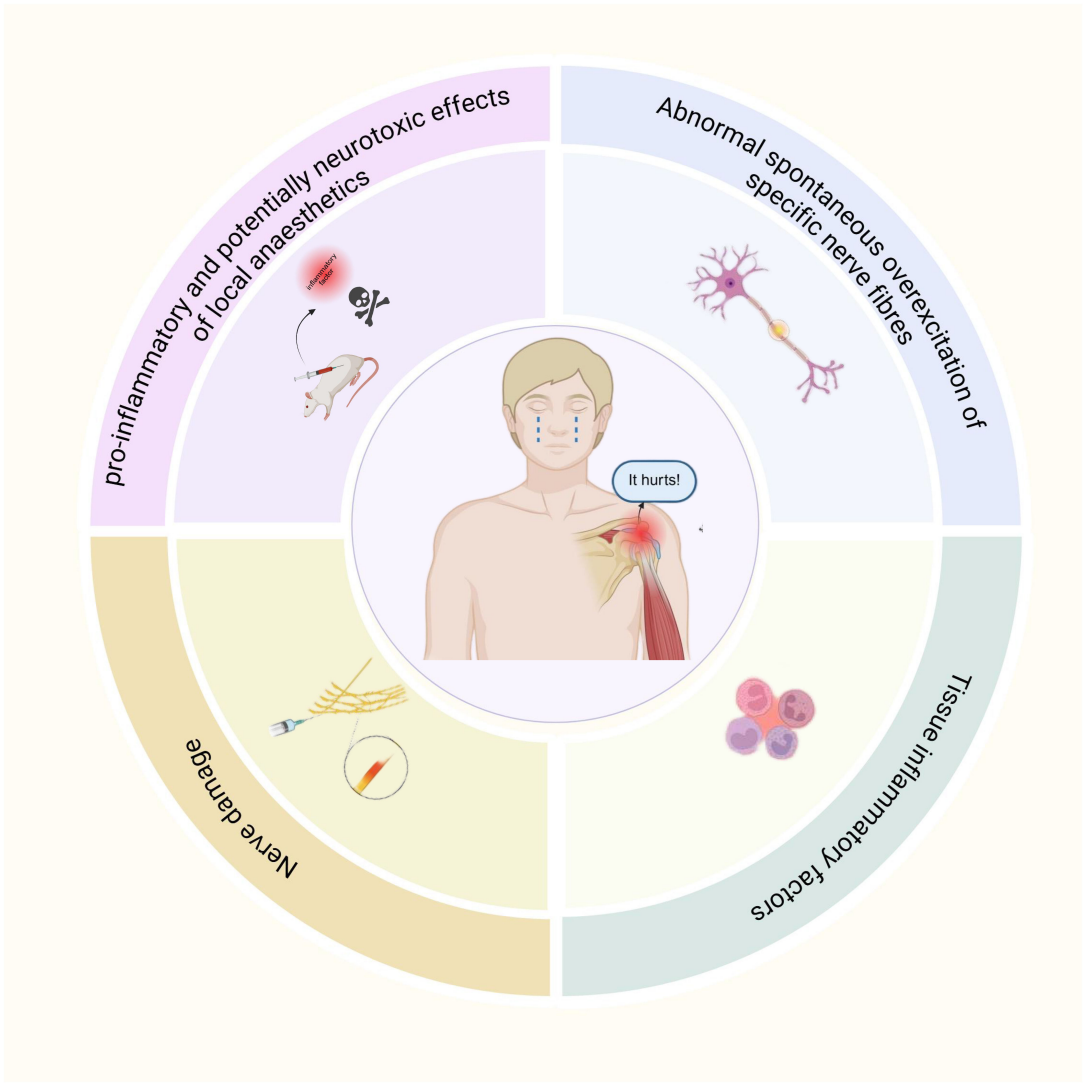
The mechanism of rebound pain.

At present, the mechanism of rebound pain after nerve block remains incompletely understood; current evidence indicates that it may result from a complex interplay of multiple factors (Figure 1).

### 4.1 Abnormal spontaneous overexcitation of specific nerve fibers

Rebound hyperalgesia following nerve block may result from abnormal spontaneous excitation in specific nerve fibers. A study demonstrated that sciatic nerve block with ropivacaine in a rat model induced transient thermal hyperalgesia after analgesia, without persistent changes in mechanical sensitization or evidence of nerve injury during a 14-day observation period (13). This observation suggests that rebound pain shares features with neuropathic pain, potentially driven by spontaneous hyperexcitability of abnormal nerve fibers and heightened injury receptor excitability in the absence of mechanical nerve injury. Anatomically, patients experiencing rebound pain are typically in a denervated state. Following a primary lesion affecting a neuron's cell body or postganglionic axon, the postsynaptic membrane of adjacent neurons may generate spontaneous discharges when exposed to local transmitters, a process referred to as “denervation hypersensitivity” (14).

### 4.2 Pro-inflammatory and potentially neurotoxic effects of local anesthetics

In clinical practice, amide-linked lidocaine hydrochloride, long-acting bupivacaine hydrochloride, and ropivacaine are widely used for anesthesia and analgesic nerve blocks in ARCR. In a rat model, perisciatic injection of ropivacaine caused degeneration of myelinated nerve fibers and axons, accompanied by increased levels of the inflammatory cytokines interleukin-6 (IL-6) and interleukin-1β (IL-1β) (15). This finding suggests that, although nerve blockade reduces nociceptive signaling, local inflammatory responses persist and may even be exacerbated. Clinical investigations have demonstrated that amide and ester local anesthetics can induce neurotoxicity through mechanisms such as DNA damage, mitochondrial dysfunction, and neuronal apoptosis (16). Furthermore, bupivacaine has been shown to increase the production of reactive oxygen species (ROS) and upregulate cellular autophagy (17). The combined effects of these mechanisms may play a critical role in neurotoxicity and could contribute to rebound pain following nerve block. Gordon et al. (18) further indicated that local application of bupivacaine and lidocaine produced pro-inflammatory effects after tissue injury, leading to upregulation of cyclooxygenase-2 (COX-2) gene expression and the increased secretion of prostaglandin E2 (PGE2), thereby promoting inflammatory pain. Conversely, Martin et al. suggested that local anesthetics may also exert anti-inflammatory effects (19). In that study, patients undergoing knee arthroplasty were divided into two groups. The control group received intravenous morphine for patient-controlled analgesia after general anesthesia, whereas the experimental group received a continuous femoral nerve block with 0.2% ropivacaine. Results showed that the experimental group exhibited significantly smaller increases in local skin temperature and knee joint circumference compared to the control group. These findings suggest that local anesthetics may exert anti-inflammatory effects through the nerve block pathway. This dual role highlights the complexity of local anesthetics in rebound pain mechanisms. Therefore, further studies are warranted to clarify the relationship between rebound pain and the pro-inflammatory effects of local anesthetics.

### 4.3 Nerve damage

Peripheral nerve block procedures and surgical interventions pose a potential of iatrogenic nerve injury. Ultrasound-guided nerve blocks provide greater safety and accuracy compared with traditional techniques. However, factors such as anatomical variations (e.g., aberrant nerve courses), excessive subcutaneous fat in obese patients affecting image resolution, operator inexperience, and other variables may contribute to nerve damage. Neurovascular compromise during nerve block may also lead to peripheral nerve injury, potentially caused by direct trauma, vascular occlusion, or intraneural hematoma (20). Anatomical studies have shown substantial variability, up to 50% or more, in the composition, branching, and alignment of the brachial plexus, with reported nerve injury rates as high as 3.16% for interscalene brachial plexus blocks (21, 22). Consequently, to reduce the risk of iatrogenic nerve injury, anesthesia and analgesic nerve block for ARCR are best performed under real-time ultrasound guidance, supplemented by nerve stimulation. During ARCR, prolonged positioning (e.g., lateral or beach chair position) and surgical manipulation may cause local nerve compression. Furthermore, patients with pre-existing peripheral neuropathy are at higher risk of nerve injury (23).

### 4.4 Tissue inflammatory factors

Rotator cuff injuries commonly result from acute trauma or chronic strain. Preoperatively, chronic ischemia and hypoxia of periarticular tendons, fascia, and other soft tissues are often present, leading to aseptic inflammation. Intraoperatively, mechanical friction from arthroscopic instruments, tissue edema, the inflammatory response induced by the saline infusion, and the local foreign body reaction to suture anchors may contribute to local tissue injury. These processes initiate a local inflammatory cascade, involving the activation of multiple inflammatory mediators and peripheral nociceptors (6).

Beyond peripheral mechanisms, central immune responses such as microglial activation also play a critical role in rebound pain. Yamada et al. (24) demonstrated that peripheral nerve block can exacerbate the acute inflammatory response following surgical incisions and other trauma. They observed neutrophil and macrophage infiltration and upregulation of PGE2 and tumor necrosis factor-α (TNF-α) near the incision site. Thus, during the recovery phase of a nerve block, marked infiltration of inflammatory cells and elevated inflammatory mediators in the surgical incision area amplify the afferent impulses of nociceptive signals. Microglia, as key components of the intrinsic immune system of the central nervous system, are the earliest responders to nerve injury (25). They rapidly react by altering their phenotype and function, secreting multiple inflammatory mediators, and establishing close interactions with neuronal cells, thereby promoting the development of pain (26).

## 5 Risk factors for rebound pain after ARCR nerve blocks

### 5.1 Surgical anesthesia factors

ARCR, a procedure within the realm of upper extremity joint orthopedics, typically uses regional nerve block, general anesthesia, or a combination thereof for anesthetic and analgesic management. The increasing use of ultrasound guidance, along with the demand for preemptive analgesia, patient comfort, and effective postoperative pain control, has promoted the widespread adoption of combined regional nerve block and general anesthesia (27). Barry et al. (28) reported that rebound pain was 6.5 times more common after orthopedic procedures than after soft tissue surgeries, with the knee and shoulder being particularly susceptible. De Boer et al. (29) found that rotator cuff repair was associated with a higher incidence of severe pain compared with other shoulder surgeries. Furthermore, postoperative rebound pain occurs more often after upper extremity surgery than after lower extremity procedures (30). In a study of patients undergoing internal fixation of distal radius fractures, those who received an interscalene brachial plexus block exhibited significantly higher pain scores at 12 h and 24 h postoperatively compared with those who received general anesthesia, suggesting that severe rebound pain may have occurred during recovery from the brachial plexus block (31).

### 5.2 Patient factors

Rebound pain has been associated with age, gender, preoperative pain level, and psychological factors (11, 32). Barry et al. (28) observed that approximately half of the patients undergoing ambulatory surgery with nerve block developed rebound pain, and their findings suggested that it was linked to patient age, gender, and other factors. The incidence and intensity of rebound pain were higher in younger patients than in older patients. This difference may be explained by diminished postoperative nociception in older patients, which contributes to greater pain tolerance and slower peripheral nerve conduction (33). Yang et al. (34) demonstrated that female patients were more susceptible to rebound pain. Female patients required higher doses of analgesic medication than males to achieve adequate postoperative analgesia. This phenomenon may be related to psychosocial complexity and sex-based differences in pain perception and experience. Furthermore, Riley et al. (35) found that patients with preoperative pain were more likely to develop postoperative rebound pain, suggesting a potential link to central and peripheral sensitization induced by preoperative pain. López-Millán et al. (36) proposed that preoperative chronic moderate-to-severe shoulder pain is a risk factor for postoperative shoulder pain, as these patients have a significantly higher risk of severe postoperative pain compared with the general population. Patients undergoing ARCR often present with preoperative pain, and those with chronic rotator cuff injuries usually experience a longer duration of symptoms.

In conclusion, patients undergoing ARCR with nerve block are at an increased risk of postoperative rebound pain. This association warrants confirmation in multicenter clinical studies with larger sample sizes. Risk factors are summarized in Table 1.

**Table 1:** Risk factors for rebound pain after ARCR nerve blocks.

**Risk factors**	**Author**	**Study type**	**Reference**	**Level of evidence**
Orthopedic procedures	Barry et al.	Retrospective cohort study	(28)	2b
	Ip et al.	Network meta-analysis	(32)	1a
Younger patients	Barry et al.	Retrospective cohort study	(28)	2b
	Yang et al.	Network meta-analysis	(34)	1a
	Ip et al.	Network meta-analysis	(32)	1a
Female patients	Barry et al.	Retrospective cohort study	(28)	2b
	Yang et al.	Network meta-analysis	(34)	1a
The absence of perioperative i.v. dexamethasone use	Barry et al.	Retrospective cohort study	(28)	2b
Preoperative pain	Yang et al.	Network meta-analysis	(34)	1a
	Ip et al.	Network meta-analysis	(32)	1a
Psychological factors (anxiety, depression)	Barry et al.	Retrospective cohort study	(28)	2b
	Yang et al.	Network meta-analysis	(34)	1a
	Ip et al.	Network meta-analysis	(32)	1a

## 6 Preventive measures for rebound pain after nerve block for ARCR

Given the mechanisms and risk factors of rebound pain following nerve block, preemptive strategies are essential. The management of rebound pain represents a multifaceted perioperative challenge that requires collaborative efforts among anesthesiologists, surgeons, and patients and involves the following interventions (Table 2).

**Table 2:** Preventive measures for rebound pain after nerve block for ARCR.

**Preventive measures**	**Reference**	**Study type**	**Author**	**Advantages**	**Disadvantages**	**Level of evidence**
Continuous nerve block technique	(39)	Meta-analysis	Richman et al.	Reduce opioid consumption and associated side effects, accelerate postoperative recovery, and provide superior postoperative pain relief.	–	1a
	(49)	Randomized controlled trial	Kim et al.	Reduce the duration of rebound pain and alleviate the severity of pain.	–	1b
	(51)	Meta-analysis	Boin et al.	–	Possible catheter tip placement errors, displacement, or dislodgement.	1a
	(52)	Systematic review	Nicolotti et al.	–	May cause infection.	1a
Combined peripheral nerve block	(55)	Meta-analysis	Zhao et al.	Provide superior analgesia and patient satisfaction while reducing the incidence of respiratory distress.	–	1a
	(56)	Randomized controlled trial	Lee et al.	Reduce the incidence and severity of rebound pain.	–	1b
Adjuvant of local anesthetic drugs	(58)	Meta-analysis	Xuan et al.	Extend the duration of nerve block.	–	1a
	(59)	Randomized controlled trial	YaDeau et al.	Enhance analgesic effects, reduce postoperative opioid consumption, and lower the risk of nausea and vomiting.	–	1b
	(61)	Randomized controlled trial	Woo et al.	Reduce the incidence and severity of rebound pain.	–	1b
	(71)	Systematic review	De Cassai et al.	–	May cause neurotoxicity.	1a
Multimodal analgesia	(73)	Systematic review	Pual et al.	Enhance analgesic effects, reduce opioid consumption, and minimize adverse reactions.	–	1a
Patient education	(75)	Randomized controlled trial	Çalişkan et al.	Alleviate postoperative patients' fear of pain Relieve postoperative pain.	–	1b

### 6.1 Continuous nerve block technique

The shoulder joint receives innervation from the cervical and brachial plexus. Cutaneous sensation of the shoulder is mainly mediated by the supraclavicular, axillary, and lateral brachial cutaneous nerves. The joint capsule is predominantly innervated by the axillary and suprascapular nerves (37). Commonly used nerve blocks in the perioperative management of shoulder surgery include brachial plexus block, superior trunk block, suprascapular nerve block, and axillary nerve block. These nerve block techniques offer effective analgesia for ARCR. However, the duration of analgesia from a single nerve block is limited. Continuous nerve block techniques can prolong postoperative analgesia, thereby reducing opioid requirements and mitigating rebound pain after nerve block administration (38, 39).

#### 6.1.1 Continuous interscalene brachial plexus block

The brachial plexus, comprised of the C5–C8 nerve roots and the anterior ramus of T1, is the target of the interscalene brachial plexus block (40). This technique effectively blocks the subscapular, axillary, and lateral thoracic nerves, thereby covering the innervation of the shoulder joint and surrounding structures. As a result, comprehensive anesthesia of the shoulder joint capsule, subacromial bursa, coracoclavicular ligaments, and cutaneous sensation of the shoulder is achieved, providing adequate analgesia for shoulder surgery (41). Interscalene brachial plexus block, often combined with general anesthesia is a preferred regional anesthetic technique for shoulder arthroscopy, as it reduces anesthetic drug requirements and provides significant benefits in postoperative analgesia and opioid sparing (42). Kim et al. (43) investigated the analgesic effects of single-injection vs. continuous-infusion interscalene brachial plexus block with an indwelling catheter in patients undergoing ARCR. The single-injection group demonstrated significant early postoperative analgesia, with lower pain scores at 6 h, followed by a subsequent increase after 12 h. Conversely, the continuous-infusion group exhibited progressively decreasing pain scores over 24 h. These findings suggest that single-injection interscalene brachial plexus block may be associated with rebound pain, likely due to the resolution of complete neural blockade.

Early postoperative mobilization of the affected limb is crucial to prevent joint stiffness and to promote functional recovery. While continuous interscalene brachial plexus block provides effective analgesia after shoulder arthroscopy, prolonged motor blockade may hinder mobility and delay recovery. Furthermore, local anesthetic may spread to the phrenic nerve region, posing a risk of phrenic nerve dysfunction, rendering this technique unsuitable for patients with respiratory compromise (44).

#### 6.1.2 Continuous upper trunk nerve block

Burckett-St. Laurent et al. (45) proposed the upper trunk nerve block to reduce phrenic nerve impairment associated with conventional brachial plexus block. The upper trunk of the brachial plexus is formed by the union of the C5 and C6 nerve roots. Peripheral nerves supplying the shoulder joint arise distally from the upper trunk, and local anesthetic injection around the upper trunk can therefore provide shoulder analgesia. Kang et al. (46) confirmed that the analgesic efficacy of the superior trunk nerve block is comparable to that of the interscalene brachial plexus block, while better preserving phrenic nerve function. Kim et al. (47) further demonstrated that continuous upper trunk block provides superior postoperative analgesia compared with continuous suprascapular nerve block in patients undergoing shoulder arthroscopy. However, further investigation is warranted to clarify the impact of continuous upper trunk block on rebound pain following ARCR nerve block.

#### 6.1.3 Continuous suprascapular nerve block

The suprascapular nerve, which originates from the upper trunk of the brachial plexus, provides essential innervation to the shoulder joint (48). Suprascapular nerve block carries minimal intraoperative and postoperative risks, making it a common technique in shoulder arthroscopy. Kim et al. (49) introduced a novel postoperative pain management strategy involving arthroscopic placement of a catheter around the suprascapular nerve, followed by continuous ropivacaine infusion. This study hypothesized that continuous analgesia via an indwelling catheter could effectively control postoperative pain, mitigate rebound pain, and reduce opioid use. Results demonstrated a significant difference in postoperative visual analog scale (VAS) scores between the single brachial plexus block group and the indwelling suprascapular nerve catheter group from 8 to 40 h postoperatively. The latter group exhibited earlier rebound pain onset, shorter duration, and lower mean pain severity. These findings suggest that continuous suprascapular nerve block may provide superior postoperative pain control compared with single brachial plexus block. Furthermore, suprascapular nerve block does not significantly impair diaphragmatic function, rendering it suitable for patients with respiratory insufficiency.

Despite the potential benefits of continuous nerve block analgesia, catheter placement poses challenges, such as incorrect tip placement, displacement, dislodgement, and puncture-site infection, which may limit its widespread clinical application (50–52).

### 6.2 Combined peripheral nerve block

Compared with a single nerve block technique, combined peripheral nerve blocks reduce the incidence of postoperative rebound pain in patients undergoing ARCR. The suprascapular nerve innervates 70% of the shoulder joint, whereas the axillary nerve supplies sensation to the lateral shoulder. Thus, a combined suprascapular and axillary nerve block represents a viable alternative to brachial plexus block (4, 53). Although either suprascapular nerve block or brachial plexus block alone may lead to rebound pain, the combined block effectively reduces this occurrence. Lee et al. (54) demonstrated that a combined suprascapular and axillary nerve block provided superior analgesia and reduced rebound pain after shoulder arthroscopy compared with suprascapular nerve block alone. Additionally, Zhao et al. (55) reported that during arthroscopic surgery, combined suprascapular and axillary nerve blocks resulted in greater pain relief and higher patient satisfaction within 24 h postoperatively than suprascapular nerve block alone. Furthermore, compared with brachial plexus block, the incidence of dyspnoea was significantly lower. Another study by Lee et al. (56) showed that the combined use of ultrasound-guided brachial plexus block and arthroscopically guided suprascapular nerve block during shoulder arthroscopy reduced the incidence of rebound pain, delayed its onset, and decreased its severity compared with brachial plexus block alone. A study on shoulder arthroplasty further revealed that patients receiving brachial plexus block combined with intraoperative periarticular injections had significantly lower 24 h postoperative opioid consumption and pain scores compared to those receiving brachial plexus block alone (57).

### 6.3 Adjuvant of local anesthetic drugs

To optimize the efficacy of ARCR peripheral nerve block, adjuvants can be co- administered with local anesthetic via perineural injection to enhance analgesia, prolong block duration, and reduce postoperative opioid requirements, thereby decreasing the incidence of postoperative nausea and vomiting (5, 58, 59).

#### 6.3.1 Dexamethasone

Dexamethasone, a long-acting glucocorticoid, is frequently used as an adjuvant to local anesthetics in clinical practice. Its use enhances analgesia, prolongs block duration, and reduces adverse effects associated with local anesthetics. Although the precise mechanism by which dexamethasone attenuates rebound pain remains unclear, its efficacy as an adjuvant has been well established. Wei et al. (60) reported that among single adjuvants to local anesthesia, dexamethasone was superior in prolonging analgesic duration and reducing opioid requirements. Woo et al. (61) conducted a study that patients received a single brachial plexus block before shoulder arthroscopy to evaluate postoperative analgesic efficacy of ropivacaine alone vs. ropivacaine combined with dexamethasone. The study found that the ropivacaine plus dexamethasone group had a more gradual block resolution, significantly smaller increases in numeric rating scale (NRS) pain scores (4.5 ± 2.4 vs. 6.9 ± 2.2), and a lower incidence of rebound pain (37.1% vs. 82.9%) compared with the ropivacaine group. Kim et al. (62) investigated the analgesic effects of perineural dexamethasone vs. continuous analgesia with an indwelling catheter in ARCR patients. The dexamethasone group demonstrated superior analgesia within the first 6 postoperative hours, but no significant difference in visual analog scale (VAS) scores thereafter. Furthermore, the dexamethasone group required fewer postoperative analgesic and less fentanyl. These findings suggest that dexamethasone, when added to local anesthetics, can prolong nerve block duration, reduce the need for postoperative analgesic requirements, and improve pain control after ARCR. The observed effects may be attributed to dexamethasone's peripheral anti-inflammatory properties or its interaction with peripheral glucocorticoid receptors, thereby inhibiting C-fiber activity and potassium channel function (61, 63). Lee et al. (64) compared the effects of adding 5 mg dexamethasone to local anesthetics with those of intravenous administration of 5 mg dexamethasone in patients undergoing interscalene brachial plexus block. The intravenous group experienced rebound pain later than the peripheral nerve injection group and demonstrated a greater reduction in the total degree of pain exacerbation and the incidence of rebound pain. These results align with the findings of Yang et al. (65), indicating that both intravenous and perineural dexamethasone administration can effectively reduce rebound pain.

#### 6.3.2 Dexmedetomidine

Dexmedetomidine, a potent and selective α2-adrenergic agonist, has shown efficacy as an adjuvant to local anesthetics. Its mechanism of action involves binding to peripheral α2-adrenergic receptors, thereby inhibiting the release of norepinephrine from nerve terminals. This action subsequently prevents the initiation of action potentials in nerve fibers and inhibits the transmission of pain signals (15). Hwang et al. (66) compared ropivacaine plus dexmedetomidine with ropivacaine alone for interscalene brachial plexus block in patients undergoing ARCR. The study demonstrated that the dexmedetomidine group had significantly lower VAS scores within 18 postoperative hours and a significant delay in the onset of rebound pain. Additionally, plasma levels of inflammatory cytokines IL-6 and interleukin-8 (IL-8) were significantly lower in the dexmedetomidine group than in the ropivacaine-only group within 48 postoperative hours, suggesting that dexmedetomidine may suppress the systemic inflammatory response. In a study by Huan et al. (67), patients undergoing shoulder arthroscopy were randomized into two groups. One group received an interscalene brachial plexus block with dexmedetomidine and ropivacaine, whereas the control group received ropivacaine alone. The results indicated that the dexmedetomidine-ropivacaine combination significantly reduced rebound pain, prolonged the analgesia duration, and improved postoperative pain control.

#### 6.3.3 Ketamine

Ketamine, a non-selective N-methyl-D-aspartate (NMDA) receptor antagonist, has analgesic, antinociceptive, and anti-inflammatory properties. A study on anterior cruciate ligament reconstruction (ACLR) compared intravenous and perineural nerve injections of ketamine. Both methods prolonged block duration and reduced the incidence of rebound pain, although the intravenous group showed a higher rate of postoperative hallucinations (68). Zhu et al. (69) demonstrated that ropivacaine combined with ketamine, administered via brachial plexus nerve block in patients with upper limb fractures, effectively reduced the incidence of rebound pain, accelerated block onset, and prolonged block duration. Conversely, the Touil et al. (70) study found intraoperative intravenous ketamine, administered after preoperative axillary brachial plexus block, did not affect the incidence or intensity of rebound pain. These findings suggest that ketamine's impact on postoperative rebound pain depends on the route of administration. Further studies are needed to clarify its efficacy in mitigating rebound pain after ARCR nerve block.

Local anesthetic adjuvants play a significant role in clinical practice, as they enhance nerve block efficacy, prolong block duration, and reduce opioid requirements. However, the combination of local anesthetic adjuvants with local anesthetic drugs may increase the risk of neurotoxicity. Research by De Cassai et al. (71) suggested that high-dose administration of local anesthetic adjuvants in patients with neuropathy may pose risks of neurotoxicity. Therefore, when local anesthetic adjuvants are combined with local anesthetic agents, the potential neurotoxic risks should be carefully evaluated, and administration should be undertaken with caution.

#### 6.3.4 Multimodal analgesia

Multimodal analgesia is a fundamental approach in clinical pain management, in which multiple analgesic agents or techniques with distinct mechanisms are administered concurrently to enhance efficacy and reduce adverse effects (72). In the context of postoperative pain management in ARCR, multimodal analgesia that incorporates nerve blocks is essential. This typically involves a combination of oral analgesics, incisional local blocks, and non-pharmacological and reduce opioid requirements. Studies by Paul et al. (73) demonstrated that multimodal analgesia strategies reduce postoperative pain and opioid usage.

Currently used oral analgesics include acetaminophen, non-steroidal anti- inflammatory drugs (NSAIDs), calcium channel modulators (pregaba lin, gaba pentin), and tramadol. The PROSPECT guidelines based on rotator cuff repair recommend regular perioperative use of paracetamol and NSAIDs in the absence of contraindications. Meanwhile, intravenous dexamethasone prolongs the duration of interscalene brachial plexus block analgesia, reduces the need for additional analgesics, and has antiemetic properties. Opioids are reserved for postoperative rescue analgesia drugs (42).

Park et al. (74) conducted a study involving axillary nerve blocks performed without imaging guidance in ARCR patients, evaluating the impact of multimodal shoulder injections on postoperative rebound pain. The findings indicated that multimodal shoulder injections significantly reduced rebound pain after brachial plexus block. Specifically, this approach was associated with a significant reduction in postoperative pain scores and opioid consumption.

### 6.4 Patient education

The precise pathophysiologic mechanism underlying rebound pain after nerve block remains unclear, which hinders the development of effective preventative strategies. Prior to nerve block administration, patients should receive comprehensive information about the procedure's benefits and limitations, including the potential for moderate to severe pain after block resolution. Enhancing patient understanding of pain perception may improve their capacity to manage discomfort after block resolution. Galos et al. (31) proposed that preoperative pain education and counseling may improve patient awareness of rebound pain. They recommended that high-risk patients use analgesic medications proactively before block resolution to optimize prevention and control. In a randomized controlled trial, Çalişkan et al. (75) found that preoperative pain management education for orthopedic surgery patients effectively reduced fear of pain and alleviated discomfort. Shih et al. (76) suggested that preoperative education combined with postoperative follow-up may enhance' comprehension and recall of pain management strategies, improve adherence, reduce perioperative anxiety and uncertainty, and potentially mitigate the incidence of rebound pain. Furthermore, incorporating techniques such as meditation and relaxation training may reduce anxiety and improve pain perception (77).

## 7 Conclusion

Nerve block is a frequently employed adjunct for anesthesia and analgesia in ARCR. However, a subset of patients experience significant pain after block resolution, a phenomenon that is frequently overlooked. This paper provides a comprehensive overview of the pathophysiological mechanisms, risk factors, and strategies for the prevention of rebound pain. However, it should be noted that, as a narrative review, this study has limitations, including the lack of a systematic literature search and the absence of quantitative data analysis. Although various strategies have been proposed to mitigate rebound pain, each has inherent limitations. Patients undergoing ARCR represent a high-risk group for rebound pain. Future research should focus on elucidating the underlying mechanisms and developing preventative and therapeutic interventions for ARCR patients. These efforts should include optimizing nerve block techniques, selecting appropriate local anesthetics and adjuvants, and designing multimodal analgesia protocols. Multicenter studies with large sample sizes are essential to reduce the incidence of rebound pain and improve postoperative pain management after ARCR.
